# Spontaneous state switching in realistic mean-field model of visual cortex with heteroclinic channel

**DOI:** 10.1186/1471-2202-12-S1-P175

**Published:** 2011-07-18

**Authors:** Manh Nguyen Trong, Ingo Bojak, Thomas R Knösche1

**Affiliations:** 1 Max Planck Institute for Human Cognitive and Brain Sciences, 04103 Leipzig, Germany; 2 Institute for Biomedical Engineering and Informatics, Technical University of Ilmenau, 98693 Ilmenau, Germany; 3 Donders Centre for Neuroscience, Radboud University Medical Centre, 6500 HB Nijmegen, The Netherlands

## 

Spontaneous switching between cortical states in the visual cortex of cat was reported by Kenet *et al.*[1]: a succession of spatial activation patterns normally associated with visual input was observed even in the absence of external input**.** Using a Wilson-Cowan network, Blumenfeld *et al.*[2] proposed a model for this phenomenon that generated multistability by applying unstructured noise. Here we use the biologically realistic mean-field model of Jansen & Rit [3], together with the heteroclinic channel theory proposed by Rabinovich *et al.*, cf. Ref. [5], to propose a mechanism how such spontaneous switching between states could occur independent of extrinsic noise.

A hypercolumn in V1 is made up of orientation preference columns (OPC), which selectively respond to specifically oriented stimuli. Our model of an OPC consists of 3 neuronal populations: pyramidal neurons (PN) and excitatory (Ex. IN) / inhibitory interneurons (Inh. IN), see Fig. 1A. Their connectivity decays exponentially with orientation difference, see Fig. 1B. These decays, and the spatial layout shown in Fig. 1C(I,II), are derived from the data of Gilbert & Wiesel [4]. The interactions between the OPCs are described by integral differential equations:

[Θ: 2^nd^ order differential operator, **V:** membrane potentials, **W:** connectivity, **S**: sigmoid function, **I:** input, **K**: gain]

Evoked activity was simulated by applying input to a specific hypercolumn, yielding patterns that are very similar to the OPC distribution maps - compare Fig. 1C(Evok.) with 1C(*IV*,*V*). Importantly however, even without any external stimulus the system spontaneously switches from one state to another, see Fig. 1C(*Spon.*). In state space the system evolves in a heteroclinic channel, made up by the trajectories near a chain of saddle points (representing the OPCs) and associated unstable separatrixes. The inhibitory connectivity governs this sequence of activation. Imposing noise on this connectivity can introduce randomness into the sequence of activation.

In this study we have combined mean-field and heteroclinic channel theory in order to describe the experimental observation of spontaneous state switching [1]. In contrast to Ref. [2], we do not need to impose unstructured noise to create multistability here. Furthermore, manipulations of our inhibitory connectivity matrix can vary the resulting sequence of states, e.g., in order to accommodate expectations about the next stimulus.

**Figure 1:**
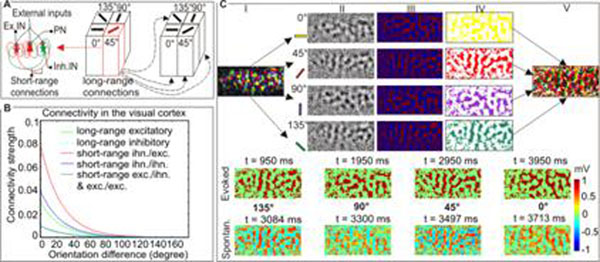
**A.** Basic model setup. **B.** Assumed decay of connectivity with orientation difference [4]. **C.** Spatial layout of OPCs and examples of the simulated evoked and spontaneous activity.

## References

[B1] Kenet T Bibitchkov D Tsodyks M Grinvald A Arieli A Spontaneously emerging cortical representations of visual attributes Nature 2003 425 954 956 10.1038/nature02078 14586468

[B2] Blumenfeld B Bibitchkov D Tsodyks M Neural network model of the primary visual cortex: from functional architecture to lateral connectivity and back J Comput Neurosci 2006 29 219 241 10.1007/s10827-006-6307-y PMC2784503 16699843

[B3] Jansen BH Rit VG Electroencephalogram and visual evoked potential generation in a mathematical model of coupled columns Biol Cybern 1995 73 357 366 10.1007/BF00199471 7578475

[B4] Gilbert CD Wiesel TN Columnar specificity of intrinsic horizontal connections and corticocortical connections in cat visual cortex J Neurosci 1989 9 2432 2442 2746337 10.1523/JNEUROSCI.09-07-02432.1989 PMC6569760

[B5] Afraimovich VS Rabinovich MI Varona P Heteroclinic contours in neural ensembles and the winnerless competition principle Int J Bifurcat Chaos 2004 14 151 158

